# F137. INFECTIONS OF THE CENTRAL NERVOUS SYSTEM AS A RISK FACTOR FOR MENTAL DISORDERS AND COGNITIVE IMPAIRMENT: A NATIONWIDE REGISTER-BASED STUDY

**DOI:** 10.1093/schbul/sby017.668

**Published:** 2018-04-01

**Authors:** Emilie Pedersen, Ole Köhler-Forsberg, Merete Nordentoft, Liselotte Petersen, Michael Eriksen Benros

**Affiliations:** 1 Mental Health Centre Copenhagen, University of Copenhagen; 2 National Centre for Register-based Research, Aarhus University

## Abstract

**Background:**

Infections and inflammatory diseases have long been suggested as risk factors for cognitive decline and mental disorders, most notably schizophrenia and affective disorders. However, largescale studies have been lacking. This study aims to investigate the association between specific CNS-infections and the risk of developing mental disorders, and whether the causal agent of the CNS-infection has an effect on the association. Furthermore, this study will investigate the possible effect of CNS-infections on cognition in the largest study to date.

**Methods:**

We will utilize the unique personal registration number to link nationwide Danish registers in order to identify all individuals born in Denmark between January 1, 1977, and December 31, 2010, with follow-up from birth. We will investigate the association between CNS-infections with the risk of 1) developing mental disorders and 2) affected cognition (defined as the highest completed level of education, completion of the 9th grade and grade average score at the end of the 9th grade). Further analyses will estimate the risk within every psychiatric diagnostic category based on the International Classification of Diseases, 10th edition (ICD-10), e.g. organic mental disorders (ICD-10: F00-09), substance abuse disorders (ICD-10: F10-19) and schizophrenia spectrum disorders (ICD-10: F20-29). The risk related to the different pathogens causing the CNS-infection will also be investigated. Data will be analysed using survival analysis to approximate relative risks estimated by Poisson regression, and will be adjusted for age, sex, calendar year, first-born status, parental history of mental disorders and educational level of the parents.

**Results:**

All analyses are expected to be completed no later than February 2018 and ready to be presented at the conference in April 2018.

**Discussion:**

This population-based cohort study will be the largest to date investigating the association between CNS-infections and mental disorders, and whether there’s a difference in risk depending on the pathogen responsible for the CNS-infection. Additionally, it will be one of the largest studies investigating the effect of CNS-infections on cognition. It will add important knowledge to our understanding of the association between CNS-infections and mental disorders, and between CNS-infections and cognition.

